# Woman in Her Psychological Relations

**Published:** 1851-01-01

**Authors:** 


### Art. II.—WOMAN IN HER PSYCHOLOGICAL RELATIONS.

The relations of woman are twofold; material and spiritual—corporeal and moral. By her corporeal nature she is the type and model of Beauty; by her spiritual, of Grace; by her moral, of Love. A perfect woman is indeed the most exalted of terrestrial creatures—physically, mentally, morally. The most profound philosophy, and the most universal instincts of the popular mind concur in this doctrine, each in their own way. The sage, the poet, the painter, see in woman the type of excellence; the mirror of the divinest attributes of the Deity; the model of the good and the beautiful—the *τὸ εῦ καὶ καλῶς*. Hence it is that she has always inspired genius. Milton gloriously writ how man's "fair large front and eye sublime declared *absolute rule*;" but of woman, that "grace was in all her steps, Heaven in her eye; in every gesture dignity and love." The ancient Greek philosophers included not merely power and wisdom, but love in their idea of God; the latter being the highest of the divine attributes, and typified by woman as creating love. It is from an obscure instinctive perception of the same idea that maternal love is typified by the ardent imaginations of the inhabitants of southern Europe, under the figure of the Virgin and Child; the unselfish, self-sacrificing love of the maternal instinct, waking up in them a sense of the sweetest, highest attribute of the divine mind, and commingling, although imperfectly, with the fundamental doctrine of Christianity, the love of God to man.

If we look at the position which woman holds in creation, and the ends which she has to fulfil to complete the designs of the Creator, we see at once that love necessarily constitutes the moving spring of a large portion of her actions, and assimilates itself with almost every motive. Upon her devolves the great duty of perpetuating the human race; and in fulfilment of this duty her feelings oscillate between man and the offspring she bears. Her "desire is to her husband;" but in common with every female animal, her feelings are concentrated upon her tender offspring; and thus it happens that, during the whole of the period in which the reproductive functions are in activity, love of one kind or the other is the ruling passion, and so her whole nature is imbued with love.

But as the physical and terrestrial only shadow forth the spiritual, so these corporeal affections in the sex are but the types of that higher and more fervent emotion, which fills the whole soul of woman when devoted to religion; and which, indeed, in virtue of that inscrutable chain that links every quality of our nature with each other, is closely dependent on, or at least intimately connected with the grosser, and corporeal passion. Often, nay, in by far the greater majority of cases, they are all commingled; the earthly feeling once excited never ceases to tint the heavenly; even in the cell and the cloister the daily routine-prayer for grace, and pardon, and help, is not inspired by a contracted selfishness, until the reproductive organs have long ceased to influence the organism, and the corporeal feelings thence resulting no longer tinge the thoughts and actions.

So much for the spiritual and moral graces of womanhood. In the corporeal we distinguish between the *beautiful* and the *pleasing;* but in both respects it is during the period of activity of the reproductive organs, peculiar to her physical construction, that the frame of woman is most pleasing and most beautiful. The excitation by woman of the mere instinct of love—the sexual passion—renders her *pleasing* in the eye of man; and this occurs, if some or all of the sexual characteristics be duly developed in her; but it is her perfect form and movement which excites his *admiration,* to the exclusion of the mere instinctive feeling. The Apollo Belvidere may "declare absolute rule:" the Venus de Medicis sets forth the grace and dignity of woman in every contour. The comparison which has been instituted by philosophic sculptors and painters, between the two famous models of human beauty, has an interesting bearing on the psychology of the sexes. It is in that portion of the body in immediate connexion with those parts peculiar to her organization, that the greatest beauty of form is found in woman, as though they were the *fons et origo* of corporeal as well as mental loveliness. "The width of the pelvis in woman causes the obliquity of the thigh-bones; the thigh therefore slopes much more inwards in woman than in man; the knee-joint is prominent on the inner side of the limb, and the graceful line limiting the thigh externally, which is strongly hollowed out on a level with the hip-joint, becomes afterwards elevated and rounded on the outer side of the leg. This inclination of the thigh on the pelvis, and of the leg on the thigh, which would constitute an imperfection in man, and a subject of mockery, gives to woman a peculiar charm. Nature ever lavishes her favours on woman in respect of forms; in her the outlines are always undulating and full of grace and suppleness; no stiffness, sharp, angular projecting masses, lines straight and meagre; the thigh, strong and powerful at its base, where it is in contact with that of the opposite side, gradually becomes more slender as it approaches the delicately-formed knee; to this succeeds the swelling of the calf, and the line of the tibia. The lower part of the limb has a grace and beauty, too well known to require any eulogy on my part; add to these the malleoli or ankle projection of a child, a small foot, most tastefully arched, a venous network, increasing by contrast the marvellous whiteness of the skin, and you will have traced the enchanting *tout ensemble* of the limbs in woman."* The same writer in describing the pelvis itself including the posterior surface of the torso, thus again supports the idea we have advanced. "The breadth of the pelvis is remarkable in woman, nevertheless its transverse diameter is generally inferior to that of the shoulders, which it sometimes equals; the haunches project outwards, but are harmoniously rounded. The contours of the back are of the most admirable purity; the region of the kidneys is elongated, the scapulæ scarcely visible; the loins grandly curved forwards, the haunches prominent and rounded; in short, the posterior surface of the torso in woman is unquestionably the *chef d'œuvre* of nature."†

It may be questioned, however, whether the glorious development of the Divine Idea in the encasing of the procreative organs and centre of procreative activity be not equalled by the bust on which the organs for nutrition of the tender offspring are developed. It is to her bosom that woman instinctively clasps all that she rightly loves—her bosom, remarkable for the unsurpassable beauty of its voluptuous contours and graceful inflexions, the white transparent surface of which is set off with an azure network, or tinged with the warm glow of the emotions and passions that make it heave in graceful undulations. The pelvis is the manifestation of the instinct—the bust expresses the sentiment of love; within the recesses of the one the embryo man is conceived and nou-

* Library of Illustrated Standard Scientific Works, vol. vii. p. 190.—(Fau and Knox's Anatomy of the external Forms of Man.)

† Ibid. p. 153.

rished; upon the other, whether babe or adult, he is hushed to slumber or soothed in suffering.

It cannot be denied, however, that rarely, if ever, is the ideal perfection of the Divine mind attained; here or there some imperfection mars the grand design; the mind of woman, or the body, or both suffer deformity. Yet we cannot but think that the most beautiful and perfect, physically, are the most excellent and perfect mentally; and that, when the two excellencies fail to be combined in the same person, the failure arises from some morbid reaction of the corporeal organs on the nervous system, or from some bias in the formative effort of the whole. It is in this respect, indeed,—the psychological imperfections in their relation to corporeal disorder and defect—that woman presents the most interesting problems for inquiry and solution; and it is only by a wide and comprehensively philosophica inquiry in the two directions indicated, that anything like a sat[illegible]actory comprehension of the problems can be acquired, or the problems themselves adequately solved.

The outline of Dr. Laycock's plan of inquiry has evidently been sketched with reference to these important guide-marks. Man is not an isolated being in creation. He forms, indeed, a part of the grand design of the Creator of such great importance—he is so manifestly made in the Divine likeness—he is so clearly at the climax of a gradual ascending scale of terrestrial life—that to separate him from that mighty system of living, feeling, active organisms, in any inquiry, whether physiological or psychological, is to depreciate rather than detract from the dignity of his nature. Being "made a little lower than the angels," it is not too much to assume that the greater and greater perfection manifested in the ascending series of animals is but the Divine plan to perfect human development; and that it originates in the Will of the Creator that man should be the most perfect of all. Nor, looking at the UNITY of created life, is it unreasonable to think that the common germ out of which the whole circle of animated beings is developed—including man —was originally made to contain, *potentially*, all the excellencies and perfections of man's nature; just as the embryo human germ—while passing through transitory phases of lower permanent animal life—still contains within it, potentially, every line and curve, and colour, which constitutes in their totality the perfect adult man. But if the human system thus contains within it, as in a microcosm, all the powers, properties, and faculties of the lower animals of the scale, it contains them, potentially, in a retrograde as well as a progressive sense. Hence it is, that we are justified by the strict application of the fundamental principles of development in looking for morbid states of the body and mind in man in the permanent states of lower animals; and we shall find, that by applying this principle of inquiry which Dr. Laycock has adopted to the psychology of woman, we can explain much that is eccentric and startling in her nature.

The anatomy and physiology of woman, in outline at least, must then have our first attention. In the embryo—to begin with the beginning—there is no difference of sex apparent, at least in the first weeks of life. It is only after the early stages of development have been gone through that a difference of sex can be traced. Immediately after birth the *general* characters of the sexes are so similar that it is only by weight and measure, or the judgment of an experienced eye, that it is possible to name from these the sex of the infant. As age advances, the general characters become more obvious, and by the seventh year the boy may be readily distinguished from the girl. He is bold, combative, muscularly active; she is retiring, timid, yielding. By the fourteenth year, the special evolution of the reproductive years has made a considerable advance, and the characteristic peculiarities of the adult human male and female are developed. In man the beard appears, the larynx enlarges, the voice deepens, the thorax expands, and is more or less hirsute on the surface. The mind matures, the intellectual powers show a different kind of activity, and the feeling of attraction for the opposite sex is more or less manifested. In woman also the voice changes, but it becomes rather mellower than deeper in tone; more pathetic and more touching in its expression. The hair grows more luxuriantly, the surface of the body is rounded from the deposit of fat beneath the skin, the skin itself is clearer softer, and smoother, and the mammæ (which are cutaneous glands) enlarge. There is also increased development of the thorax, but less than in man; the pelvis being more developed in woman. The mind undergoes a corresponding change; the perceptive faculties being, however, more developed than the intellectual. It is in virtue of this that woman enjoys that greater insight into character, and that almost instinctive perception of motives, which she possesses, and which is often concealed under an appearance of charming artlessness and modesty. Cabanis, in his "*Rapports du Physique et du Moral de l'Homme,*" elegantly describes this instinctive acuteness of the perceptive faculties: "Elle doit se réserver aussi cette partie de la philosophie morale, qui porte directement sur l'observation du cœur humain et de la société. Car vainement l'art du monde couvre-t-il et les individus, et leurs passions, de son voile uniforme: la sagacité de la femme y démèle facilement chaque trait et chaque nuance. L'intérêt continuel d'observer les hommes et ses rivales, donne à cette espèce d'instinct une promptitude et une sûreté que le jugement du plus sage philosophe ne saurait jamais acquérir. S'il est permis de parler ainsi, son œil entend tous les paroles, son oreille voit tous les movemens; et, par le comble de l'art, elle sait presque toujours faire disparaître cette continuelle observation sous l'apparence de l'étourderie ou d'une timide embarras."*

There are other characteristics which we shall presently notice, but to assist us in comprehending the psychological relations of woman, we will here observe that, although there is doubtless a general difference in the constitution of the two sexes, many of the more *special* characteristics are either dependent upon the influence of the reproductive organs, or are *general* characteristics rendered more marked or exaggerated by the same influence. The development of these organs in man and animals generally corresponds very closely to the flowering of plants; and numerous interesting analogies may be traced between the adult life of flowering plants and the adult life of man. The flower is simply a terminal bud, including the organs of reproduction by seed, which are properly the stamens and pistillum. The essential part of the former is the anther, corresponding to the testes and secreting the pollen or fecundating matter; the essential parts of the pistillum are the stigma, collecting the fecundating particles, and the ovarium to which they are conveyed. This latter corresponds to the ovaria of the human species. The comparison which has been instituted by poets, between the accession of the age of puberty and the flowering of plants, is as philosophical as it is graceful. The *blooming* maiden, glorious in the *lumen juventæ purpureum*, is well compared to those brilliant flowers, the reproductive organs of which, when fully developed, are surrounded with the most gorgeous tissues—for what reason we know not. Many animals are equally adorned with ornaments, the development of which is contingent on the development of the reproductive organs. Ripe womanhood has a lustre peculiar to itself, but inferior to none. The influence of these essential organs of reproduction on the corporeal and mental characteristics of the two sexes have been traced by Dr. Laycock throughout various classes of animals, and their bearing in man and his nature (including woman) fully illustrated. Thus the colour, composition, and form, of the numerous cutaneous appendages of animals are often exclusively connected with these fundamental functions of the reproductive organs; and there cannot be a doubt that the appearance of these appendages to the opposite sex, exercises an important influence upon the sexual instinct. Usually, the male is more brilliant and more beautiful than the female; and this is particularly striking in butterflies and birds, in which (as in many flowers) the Divine Idea has lavishly displayed every possible combination of the beautiful in colour and form. Thus in the genus *Polyomatus*, the wings of the male butterfly are of a deep blue glossed with violet, while those of the female are of

* Op. cit., vol. i. p. 312.

an unpretending dark brown fringed with grey. The butterflies of the *aves*, namely birds of paradise, manifest the operation of the same general law; and not only has the male bird the most gorgeous combination of colours imaginable, while the female is clothed in humble russet, but his tail and neck feathers are arrayed in the most graceful groupings, with a perfection of art which the most skilful *plumassier* in vain attempts to imitate. In man, and the higher vertebrata, the luxuriant growth of hair on the neck, face, and thorax, constitutes the most striking cutaneous appendage of this kind. Now it is a law of nature that these sexual appendages in the male shall attract the female to him; they are supplied to the male for this express purpose, indeed the cocks of various gallinaceous birds strut about like veritable beaus when wooing, and display their figure and their feathered ornaments, to their "intended," in the most gallant and graceful manner; each threatening his rivals like a brave warrior, and displaying his energy and his readiness to do battle. On the other hand, the female, by the same law of adaptation, is so constituted, that she is pleased by the display; her sexual instinct is roused, and she yields to the attraction. With a wisdom and a foresight most admirable to contemplate, it is so arranged that if by disease, or in any other way, the essential organs of reproduction in the male be rendered imperfect, and be therefore unfit for their office, these attractive appendages are not developed, or if developed already, drop off. It is for this reason that the effeminate man is no favourite with woman. Woman, in virtue of that mysterious chain which binds creation together in one common bond of vitality, is not exempt from this influence of colour and form. Often, indeed, it is not recognised, or if recognised, its true nature and bearings are not understood; but many a scene of domestic anguish might have been averted, and many an irrevocable sacrifice prevented—the sacrifice of home, reputation, friends, conscience—to the gratification of an irresistible passion, if this secret influence of external form and colour on the mere instinct had been met and counterbalanced. The soldier is *par excellence* the most attractive to the sex; his warlike profession, his manly moustache, the scarlet and gold, the nodding plume, the burnished helm of his uniform, his glittering arms, and the tout-ensemble of his accoutrements, often, where there is a special susceptibility to the sexual influence of form and colour, awake strange mysterious emotions in the young female just bursting into womanhood, that quickly shape themselves into a longing desire, the object of which she scarcely comprehends. Different in its origin, but analogous in its nature, is the preference so often given by the more susceptible portion of the sex to the manly sensualist. The vigorous bold front, the ample beard and luxuriant hair, the broad chest, the firm port, and an eye flashing passion and admiration, too often carry away an amorous female; and she yields to the tempter, against her better judgment, in spite of the earnest entreaties of her friends, and to the utter rupture of the dearest ties—not even excepting the maternal. This *enchantment*—which it literally is—this infatuation, is often due to the unrecognised reaction of the physical appearance of the tempter upon the mind of his victim, untrained to self-control, predisposed to the allurement by an excess of reproductive energy, and irresistibly impelled forward to the gratification of the obscure, deep-felt longings he excites by an over-stimulated nervous system.

There are some sexual allurements peculiar however to man, which require notice under this head, that do not appear to be dependent upon sexual characteristics as such, and yet are singularly potent. It is a common observation that peculiarities of form and complexion (but particularly of complexion) have a special charm for the opposite sex. Thus, the dark-eyed, dark-complexioned woman prefers the man of a fair-haired race; while the fair-haired, blue-eyed man prefers the brunette. That this preference arises out of an instinctive desire implanted in man's nature by the Creator, is manifest; for it corresponds in every particular with his other instinctive desires, and when analysed, may be clearly classed amongst the sexual stimuli. When it has wholly seized and occupied the mind, it excites the most intense emotion, and is more frequently, we believe, than any other sexual stimulus, the cause of "love at first sight." That it is physical is, we think, obvious, from the circumstance that marriages resulting in a mere instinctive prepossession of this kind are not unfrequently ill-assorted, morally and socially, in consequence of the wide difference in the tempers, taste, and even innate prepossessions of the parties. If we might venture a surmise as to the object of the Creator in implanting this instinct in man's nature, in connexion with the propagation of the species, we should pronounce it to be the crossing and improvement of races; for it is well known that crosses in blood, as creoles, for example, present the most perfect types of physical beauty. The prince of amatory poets has not let the circumstance escape his notice, and has connected it with the theory of predestined matches:—

"Oh! there are looks and tones that dart
An instant sunshine through the heart,—
As if the soul that minute caught
Some treasure it through life had sought.

As if the very lips and eyes,
Predestined to have all our sighs,
And never be forgot again,
Sparkled and spoke before us then!"

*Lalla Rookh—The Light of the Harem.*

This sympathy, this *special* attraction between individuals of the two sexes, has given rise to various philosophical and popular speculations. It has been thought that there is a fate in marriages, or that marriages are made in heaven. M. F. Tupper refers obscurely to this idea in his proverbial philosophy.

> "If thou art to have a wife of thy youth, she is now living on earth;
> Therefore think of her and pray for her weal; yea, though thou hast not known her."

The ancients had a notion that man was originally androgynous, a being compounded, like a flower, of the two sexes in one; that subsequently a division took place, and as only half an individual comes into the world at each birth, under the altered circumstances, the two halves so separated seek to be united again to each other, in virtue of an imperious sympathy; and that inconstancy in love and marriage resulted from the difficulty which the two halves experienced in finding each other. A witty French writer, referring to this secret sympathy, sarcastically observes, "Une femme nous parait-elle aimable? Nous la prenons pour cette moitié avec laquelle nous n'eussions fait qu'un tout; le cœur dit: la voilà, c'est elle; mais à l'épreuve, hélas! trop souvent ce ne l'est point!"

Such a theory may have probably originated in the biblical account of the creation of woman, for we find Milton broaches the identical idea in his noble epic. Eve relates how Adam claimed her as his other half.

> "'Part of my soul, I seek thee, and thee claim,
> My other half.'—With that thy gentle hand
> Seized mine."—*Paradise Lost.*

The sense of smell participates in exciting the vital actions connected with the reproductive process to a much greater extent than is usually supposed. Sexual odours seem, however, to be more frequently excitants of the passion in males, than in the females. The virgin female of certain moths and butterflies is used by aurelians for the purpose of capturing the male insect; if the female be in a room, the window of which is left open, the male will fly in at the window, go directly to her, and so lose all sense of fear when they approach her that they may be readily taken by the hand. Dr. Laycock enumerates a variety of animals, both male and female, which have sexual odours; the most common odour is *musk* or some of its modifications. The influence of sexual odours is irresistible in various animals. The description of its operation on the horse is beautifully described by Virgil:—

> "Nonne vides, ut tota tremor pertentet equorum
> Corpora, si tantùm notus odor attulit auras?"

Trembling with amorous rage as they sniff the well-known scent, they stand a moment, then break away in headlong fury.

> "Ac neque eos jam fræna virûm, neque verbera sæva

> Non scopuli, rupesque cavæ, atque objecta retardant

> Flumina, correptos undâ torquentia montes."

The use of scents, especially those allied to the musky, is one of the luxuries of woman, and in some constitutions cannot be indulged without some danger to the morals, by the excitement of the ovaria which results. And although less potent as aphrodisiacs in their action on the sexual system of woman than of man, we have reason to think that they cannot be used to excess with impunity by most. It would appear as if their *medicinal* virtue, in various forms of female diseases, were owing to this influence on the ovaria, especially in the spasmodic class, usually acknowledged to result from continence in strong sexual women.

Musical sounds have a powerful influence on the instinct of propagation, and their production seems to accompany it and stimulate it in numerous classes of animals. In male mammals the voice is always deeper in tone and more sonorous than in the female. The male of singing-birds is alone musical; the female, as is well-known, is silent. During the moult, or when the instinct is dormant, the musical voice is dormant. The cuckoo, for example, ceases to sing musically when his parental cares are over.

> "From a fiddle out of tune,
> As the cuckoo is in June."—*Ben Jonson.*

Milton also refers to what is, doubtless, the great end of the musical performances of the male bird—the solace of his mate :

> . . . . . . . . "For beast and bird,
> They to their grassy couch, these to their nests,
> Were slunk, all but the wakeful nightingale;
> She all night long her *amorous* descant sung."

The voice in the human species has a reciprocal influence. Nothing shocks the amorous sentiments and dissipates them so much in man as a coarse, harsh voice in woman. The illusion created by the charms of the person is strangely broken, if on hearing her voice it be not in harmony with her other attractions. On the other hand, nothing is so touching and captivating in woman as a tender, loving tone of voice; and it is certain that amorous feelings modify it much. A young lady, remarkable for her musical and poetical talents—especially for tender lyrics—naïvely remarked to a friend, when complimented upon her singing, "I never sing half so well as when I've had a love-fit."

A French editor of Lavater's works—no unskilled or superficial observer—makes an interesting remark on this point: "On observe dans quelques autres voix de femme un timbre qui, sans être aussi doux, doit un effet non moins enchanteur aux dispositions tendres et amoureuses qu'il révèle. Elle est plus animée que touchante; elle a quelque chose de plus aigu, de metallique; et l'oreille d'un physiologiste ne peut y meconnaître l'exaltation vitale que les organs de l'amour impriment d'une manière sympathique, à ceux de la respiration." Shakspeare truly remarked, that a sweet voice is a pleasant thing in a woman. To the close observer, nothing is so characteristic of the temper of a woman as her voice; during the period of activity of the reproductive organs it is the sweetest; but a really sweet voice, such as accompanies a loving, gentle, forgiving temper, will long survive the climacteric period, for it is rhythmical as well as musical. But it is the influence of man's voice, and of music, on *woman*, that we have to consider. This sexual influence is clearly twofold. There is, first, the influence of the sexual voice operating alone—the deep, sonorous voice of the male man—if we may be permitted the term, and which is exactly analogous in its origin to the roar, the neigh, the bellow of other male animals. This voice will have an effect on an amorous or susceptible organization much in the same way as colour and the other *visual* ovarian stimuli, which we have already noticed. A manly voice is without doubt pleasing to a true woman, as a shrill, weak voice in a man is displeasing, especially if in other respects he be effeminate or unmanly. We believe a more important and more permanent influence is exercised by the same kind of voice when modulated to music. In this respect, man has something in common even with insects as well as birds,—namely, those which are possessed of musical instruments and play on them to attract the female. The male green field-cricket plays on a drum; the male hearth-cricket, on something like a tambourine; the male cicadæ—for, in all these instances, it is the male that is musical—

> "Happy the cicadas' lives,
> For they all have voiceless wives,"

is the observation of the Rhodian poet, Xenocritus—the male cicadæ have a sort of harp made of a pair of drums, one on each side, fixed to the trunk between the belly and hind legs, with which a bundle of muscular cords is connected; and are thereby enabled to elicit sounds not unlike those of a harp, when they seek for a female. Others of this class produce trumpet-like notes. The lover will not only serenade his mistress, but woo with woeful ballad made to her eyebrow. It is one of the pithy sayings of Lacon that, "love makes many rhymers, but few poets;" a more prosaic idea than Moore's—

> "And every sigh the heart breathes out
> Is turned, as it leaves the lips, to song!"

The kind of poetry will depend upon the education and tastes of the individual; but the principle is perhaps universal in its operation, and is another proof of the existence of that mysterious chain of formative and divine ideas which links creation together. It shows, that it is not physical beauty only which the Creator has connected with the reproductive organs. Their mysterious influence thrills through man's whole soul as well as his mere bodily organism; and gives life to the purest, sweetest, most enchanting strains of the poet, as well as to the descant of the "amorous nightingale." The practical point of all this is, that where we have a class of stimuli so generally excited, we may feel sure that the object and *recipient* of them has an organization adapted to them, and therefore, that in this case the mind of woman must be influenced sexually by the large amount of amorous poetry and music written and sung in society. Probably, it is in virtue of this characteristic of her organization that she prefers vocal music of a gentle, pathetic, simple kind, to the more refined and more scientific instrumental performances. M. Lambert, a dialogue-writer of the last century, justly observes, on this point: "Les bruits forts et les sons éclatans, qui plaisent à l'oreille de l'homme, ébranlent fortement la votre. L'harmonie qui résulte d'une grand nombre de voix et d'instrumens, plait médiocrement aux femmes; il ne leur faut qu'une musique douce et tendre, enjouée ou pathétique."

The *touch* is the last sense we shall notice as a medium by which those stimuli enter the mind of woman that wake up her sexual instincts and emotions. We believe nothing is so exciting to the instinct or mere passion as the pressure of the hand or those tactile caresses which mark affection. They are the most general stimuli in lower animals. The first recourse, in difficulty and danger, and the primary solace in anguish, for woman, is the bosom of her husband or her lover. It is by a sort of instinctive reflex *outness* that she seeks solace and protection and repose on that part of the body where she herself places the objects of her own affections. Woman appears to have the same instinctive impulse in this respect all over the world. Glorious Milton thus touches on this point in the natural history of woman:

> "So spake our general mother, and with eyes
> Of conjugal attraction, unreproved,
> And meek surrender, half embracing, leaned
> On our first father; half her swelling breast
> Naked met his under the flowing gold
> Of her loose tresses hid: he in delight,
> Both of her beauty and submissive charms,
> Smiled with superior love, . . . . . .
> . . . . . . . and pressed her matron lip
> With kisses pure."—*Paradise Lost.*

When a few years of puberty have elapsed, it is the privilege and duty of woman to be married and bear children, provided it be her lot to fulfil her destined end on earth. Previously to this, she has to receive the attentions of her lover, and to decide whether she shall accept or reject. We do not propose to give an essay on courtship and matrimony, so we pass over this interesting portion of woman's history, to notice the period when the pleasing yet anxious duties of maternal love commence. Here, again, we have a vast number of instructive analogies in the lower creation to guide us, for the Creator has assigned to the female the almost exclusive duty of providing for the corporeal wants of her offspring—in many instances for every want,—although in some the labour is shared by her mate: whilst in others, (as bees, &c.) the cares of the nursery are the duty of a sort of commonwealth. So soon as the reproductive organs take up the serious business of continuance of the species, and the pleasures of love have given place to the formation, development, and protection of the young animal, numerous changes in the mental condition take place. The mind is less directed towards the instinctive stimuli of desire; and changes in the nervous system, accompanied with corresponding changes in the temper, are observed.

The modifications of the *appetite* necessary in the females of lower animals, for the proper nutrition and development of the ovum or fœtus, are occasionally reproduced in the pregnant human female as *morbid* appetites; but perhaps they, like other similar modifications of the instincts, occur more frequently, *proportionally*, in the young unmarried female. It has been observed by naturalists that birds will eat lime or chalk while laying—obviously that the shell may be duly formed; for, if hens be deprived of the opportunity of obtaining it, the eggs have only a membranous covering, or an imperfect shell. So, also, female carnivorous animals have the appetite for their natural food more ravenously excited during utero-gestation and lactation, to the same end—namely, that of duly perfecting the nutrition of the young animal. These morbidly excited appetites are known as "longings" in the pregnant woman, and in the young unmarried woman, as *pica* and *bulimia*. This change in the appetites has always attracted popular attention, and given rise to much astonishment, but we are now enabled by Dr. Laycock's doctrines to trace them to their origin. Dr. Laycock observes, that "although during pregnancy some good wives 'long' for handsome dresses, furniture, &c., yet these longings are *spurious*, since the morbid feelings belong exclusively to the appetite for food. Ben Jonson notices these spurious longings.

"'*Littlewit.*—O yes, Win: you may long to see as well as to taste, Win: as did the 'pothecary's wife, Win, that longed to see the anatomy, Win. Or the lady, Win, that desired to spit in the great lawyer's mouth, after an eloquent pleading.'—*Bartholomew Fair*, Act iii. Sc. 1."

Ben Jonson, indeed, seems to have had some experience of this form of morbid appetite, for he refers to it again and again in his plays. Thus, in Act 1st of that just quoted, he makes the same character say—

"Win, long to eat of a pig, in the fair, do you see, in the heart of the fair, not at Pye-corner. Your mother will do anything, sweet Win, to satisfy your longing, you know; pray thee, long presently, and be sick o' the sudden, good Win," &c.

The things desired in this ovarian perversion of the appetite are sometimes very extraordinary, and outrageously absurd. Dr. Laycock quotes Dr. Elliotson as mentioning in his lectures that a "patient has longed for raw flesh" (the carnivorous appetite) "and even for live flesh, so that some have eaten live kittens and rats." Langius, a German writer, tells a story of a woman who lived near Cologne, who had such a cannibalish longing for the flesh of her husband, that she killed him, ate as much of him as she could while fresh, and pickled the remainder. Another longed for a bite out of a baker's arm! More marvellous masticators, as Dr. Laycock observes, than the "case" described by Ben Jonson, in his play of "The Magnetic Lady"—(although Dr. Laycock quotes the case of a German woman who would eat a bonbonnière of *charcoal.*)

> . . . . . "She can cranch
> A *sack* of small coal, eat your lime and hair,
> Soap, ashes, loam, and has a dainty spice
> Of the green-sickness."

This "dainty spice of the green-sickness," thus described by rare Ben, is described by pathologists under the term of "Temper Disease." It is attended by the impaired digestion and defective assimilation which characterizes chlorosis, and by the most extraordinary perversions of temper, very frequently with regard to diet; the patient persisting in a system of starvation, or only taking the most improper food, or that which she can get by stealth. Here, again, we trace a link of the mysterious chain which connects organisms together, and can have little doubt that this form of psychological change is due to a morbid action of the reproductive organs, such as occurs occasionally in pregnancy.

There are other alterations in the mental character of woman belonging to this class of perverted instincts, which are of greater importance, because they involve the social and moral relations. The hysterical *cunning* of the young female is traced by Dr. Laycock to the same ovarian source. Referring to the development of certain instincts in the female at the period of procreation, and when the care of offspring is the great end of life, he compares the artfulness of lower animals with this hysterical cunning, and attributes it to the influence of the ovaria on the nervous system. In the males of various animals—the salmon, and others among fishes—and those of the gregarious *aves* and *mammalia,*

concurrently with the periodic excitation of the reproductive organs, there is a combative propensity developed. Virgil has vigorously described the combat of the bulls of a herd.

> "Illi alternantes multa vi prœlia miscent

> Vulneribus crebris; lavit ater corpora sanguis,

> Versaque in obnixos urgentur cornua vasto

> Cum gemitu: reboant silvæque et longus Olympus."

> *Georg. Lib.* iii. v. 220.

The female, so far from being warlike, is timid, cautious, and artful, except when present violence threatens her offspring. Dr. Laycock observes that astuteness is as much the characteristic of woman as courage is of man; but that these characteristics are not morbidly developed except under given circumstances. "It is not until puberty, however, that these peculiar qualities of the constitution of woman are distinctly brought out; and in brutes it is only when the business of reproduction is carried on, that this artfulness is so exalted as to rival the highest attempts of human sagacity. The skill they display in the choice of a secret place in which to deposit their eggs, or young, and the *finesse* with which the latter are protected from discovery or injury, are well known to the most inexperienced student of natural history. The lioness, for example, ferocious and powerful as she is, when she fears that the retreat in which she has placed her cubs will be discovered, will hide her footmarks, by retracing the ground or brushing them out with her tail." When the young female suffers from irregular action of the ovaria on the system, the natural astuteness and quickness of perception degenerates into mere artfulness or monomaniacal cunning; and it is to this morbid influence of the ovaria on the organ of mind, that Dr. Laycock attributes the extraordinary instances of monomaniacal cunning in females, on record. He observes, on this head, "of all animals, woman has the most acute faculties; and when we consider how much these may be exalted by the influence of the reproductive organs, there is not much ground for surprise at the grotesque forms which cunning assumes in the hysterical female, although they have caused much speculation and astonis' ment. Insane cunning is usually exhibited in attempts at deception, but occasionally in a propensity to steal, or rather to steal slily. It may be remarked, that when it occurs, it may be as much a symptom of hysteria as any corporeal affection whatever. It is a true monomania, and is most likely to occur in the female who is hysterical from excess of sexual development—*one possessing the utmost modesty of deportment, and grace of figure and movement, for the modesty itself springs out of that feminine timidity to which I have just alluded.* Sly stealing, however, is most frequently observed in pregnant women." The italics in the above quotation are our own, as we wish to direct the the reader's special attention to the important principle pointed out by Dr. Laycock. The propensity, in such case, is dependent solely on the excitement of the nervous system by the ovaria; hence it is, that when, in consequence of an active condition of those structures, the graces peculiar to the feminine character are peculiarly developed, and gentleness, modesty, and timidity, are prominent characteristics, often in those identical cases it is, that there is this morbid excitation of the instinct of artfulness or cunning; and it is these endowments which explain the influence that hysterical girls have upon all that come near them, and which is, as Dr. Laycock observes, really "astonishing: parents, women, physicians, all yield to them." It is also the marked excitation of this sexual artfulness which renders nugatory all the experiments and labours of those mesmerists, whose principal subjects are young females or youths about the age of puberty. Psychologists, practically acquainted with this subject, can place no reliance upon the statements of the hysterical females upon whom mesmerists experiment, however well educated, gentle, good, and truth-loving they may be naturally, and really *are* in all other matters. Physicians have recorded numerous instances of strange and motiveless deceptions, thefts, and crimes practised by young women, even by ladies of unexceptionable morals, excellent education, and high rank. Fasting women, *ecstatica*, sly poisoners, pilfering lady-thieves, &c., present examples of this kind; particular instances we need not mention, as they may be found in most works on hysteria, and often occupy a niche in the newspapers. When *cunning* is combined with a morbid excitation of the propensity to destroy, such as is manifested in the females of brutes, the effect is sometimes dreadful, and is seen in the perpetration of *secret* murders by wholesale poisoning, or in secret incendiarism; and if other natural instincts be perverted, the objects of woman's warmest and most disinterested affections may perish by her hand. It is a singular fact, in natural history, and remarkably illustrative of our views, that parturient domestic animals sometimes suffer from the same morbid condition of the nervous system as the human mother, and they also destroy their offspring. Thus cats, sows, and bitches, have been known to *eat* their litter; cows to butt their calves to death, hens chase their chickens, &c. When cunning is combined with a morbid state of *the temper*, the misery inflicted upon domestic peace is inexpressible. The ingenuity in malice and falsehood displayed by the patient is most extraordinary; so extraordinary, indeed, that it is never credited until it is experienced. Cases are by no means infrequent in which the sufferer from this sad derangement is the most intellectual and most amiable of the family; beloved by all, respected, almost worshipped. Hence, when, after numerous struggles to repress them, the propensities, excited into such fearful and almost supernatural activity, by the ovarian irritation, burst forth beyond all control, and the pet of the family is seen to be the opposite, morally, in every respect to what she had been—irreligious, selfish, slanderous, false, malicious, devoid of affection, thievish in a thousand petty ways, bold—may be erotic, self-willed, and quarrelsome—the shock to the family circle and friends is intense; and if the case be not rightly understood, great, and often irreparable mischief is done to correct what seems to be *vice*, but is really *moral insanity.* Dr. Laycock, we are happy to learn, has been able to treat cases of this kind with perfect success, by a course of galvanism directed through the ovaria, and by suitable medication and moral and hygienic treatment.

Perhaps in the whole range of psychology there is no subject so deeply interesting as this; for it is in moral insanity that man's spiritual and moral nature is the most awfully and most distressingly subjected to his corporeal frame. It is a disease undoubtedly much more frequent in the sex than in man; and if the warning voice we shall here raise against all those methods of education, and mental and physical training—all those conventional customs and social habits—all those *fashions* in dress and social intercourse, which stimulate the nervous system generally of the sex, and the sexual system in particular—be at all successful in placing woman in greater safety from this sad clouding of her intellect—this lamentable spoliation of her greatest charms, we shall feel that we have done good services to society and the state.

Shall we omit the consideration of the psychology of "Old Maids?" Our gallantry forbids us; for, although their "single blessedness" may have left them to pass through the world "in maiden meditation fancy free," the non-fulfilment of their duties as *women* involves its punishment, or its penalty. Celibacy is more frequent in the middle and higher classes of society than in the lower, with whom prudential considerations have less weight; hence it is that the "Old Maid" is seldom to be found in that class. It is not difficult to trace the gradual development of the mental and corporeal peculiarities of the woman who has passed middle life in celibacy. A great void in her nature has been left unfilled, except occasionally. At first, the future victim of society's conventionalism is "as scornful as scornful can be" in the flush of youth and beauty. She expects to see "wit and wisdom and gold" at her feet, and hardly understands how it is that year after year glides away, and she is still unmarried, until she discovers, when it is too late, that pride and haughtiness mar woman's charms, however charming; and that anyhow they repel the timid lover. Then, when the climacteric period is dawning upon her, she possibly makes a foolish match, in sheer desperation, with her junior in age, her inferior in station, and her unequal companion in every respect. Or, if prudence still guides her, she lavishes the love with which her nature is instinct on nephews and nieces, or some pet family. Or the love that would have found its natural outpouring on a husband or children, may be directed by religious feelings to suffering humanity, and she may become warmly charitable; or if the intellect be contracted and selfish, it may find vent in domestic or tame animals. Hence the cat, the parrot, and the poodle, are connected popularly with arid virginity.

With the shrinking of the ovaria and the consequent cessation of the reproductive nisus, there is a corresponding change in the outer form. The subcutaneous fat is no longer deposited, and consequently the form becomes angular, the body lean, the skin wrinkled. The hair changes in colour and loses its luxuriancy; the skin is less transparent and soft, and the chin and upper lip become downy. Sometimes, indeed, the male characteristics are in part developed (a change which has been observed in lower animals to occur concurrently with a change in the ovaries) and a hoarser voice accompanies a slight development of the beard. With this change in the person there is an analogous change in the mind, temper, and feelings. The woman approximates in fact to a man, or in one word, she is a *virago*. She becomes strong-minded; is masculine in her pursuits, severe in her temper, bold and unfeminine in her manners. This unwomanly condition undoubtedly renders her repulsive to man, while her envious, overbearing temper, renders her offensive to her own sex. If there be such a change in the ovaria that the temper is modified in the way we have described, the "Old Maid" is the pest and scourge of the circle in which she moves; and in extreme cases—verging upon, if not actually the subject of—worse insanity, she is little less than a she-fiend. Her whole life is devoted to an ingenious system of mischief-making; she delights in tormenting—corporeally and mentally—all that she dare to practise upon. She is intrusive, insolent, regardless of the ordinary rules of politeness; ever feeling insults where none were intended; ungrateful, treacherous, and revengeful — not sparing even her oldest and truest friends. Add to these mental characteristics, a quaint untidy dress, a shrivelled skin, a lean figure, a bearded lip, shattered teeth, harsh grating voice, and manly stride, and the *typical* "Old Maid" is complete.

But such is not frequently the unfortunate condition of the aged, childless, mateless woman. Religious duties take the place of the domestic, and the abounding love, which she cannot lavish upon husband and children finds a more sacred outlet. When this is the case, an admirable character is the result. Self-denial and humility; an expansive, ever-active charity; candour, gentleness, and amiability; an unobtrusive goodness of heart; a love of social and domestic pleasures; these are a few of the qualities of the woman who, having failed to fulfil the great

*physical* end of her existence, has head and heart enough to see that she has also *moral* duties not less important. It is from this class that the ranks of the "Sisters of Charity" are filled up; and it is this class of women who constitute the most active agents in the "good works" of religious societies.

Perhaps we should only be doing justice to our subject if we were to extend our historical sketch, so as to include the wedded-life woman; and review her psychological relations when occupying her true position as the wife and mother. The circle of family relations—husband, father, son, brother—is to the true woman, and to all she blesses with her presence, a "perpetual fountain of domestic sweets;" blessed with these objects upon which to lavish her love, she wants none else:

> . . . . . . "to know no more
> Is woman's happiest knowledge and her praise."

Often her warm, truthful love is slighted; often Providence denies half the delight of wedded life, and she is childless. These circumstances have an important influence on her character, for good or evil; but our space will not permit this extension of our subject. We will, therefore, turn the reader's attention to some practical points in th social and domestic condition of the sex, and note especially how injudicious management and an imperfect hygiene warp the whole future life of the woman, by an injurious influence upon those deep and hidden sources of action in her nature to which we have adverted. In doing this we shall also notice, incidentally, the psychological relations of the nervous and vascular systems of the sex, and of those higher endowments which constitute the intellect.

Much attention has always been directed to the periodic modification of the health, which has been thought (but erroneously) to be peculiar to the sex; there can be no doubt that, although its bearings are often mistaken, and derangements in its recurrence are often the effect rather than the cause of disordered general health, it may, rightly interpreted, be made available by the psychologist. The susceptibility of the system to all kinds of impressions is much increased during that period; and many of those modifications in the temper and feelings to which we have referred as permanent, occur in an evanescent form during the time of periodic disorder. Hence these temporary and passing alterations in the health may often point out to us the type and kind of real disorder from which the individual may permanently suffer. In this respect, an early development of the uterine function indicates an early development of the ovaria, and therefore a degree of sexual precocity proportionate to the health of the girl. Precocious menstruation hardly ever takes place without more or less disorder of the system; and in those instances of remarkable precocity in which the flow has occurred at the early age of seven or eight years (and many such cases are on record), life has rarely been prolonged to ordinary adult age; nor in those cases in which the flow has commenced at or about the age of eleven or twelve, has the health usually been perfect; and the individual has suffered from various and extraordinary diseases of the nervous system. Hence it is very fairly inferred, that a too early development of the sexual functions leads to disease—especially of the nervous system—or, if not to any well-defined form of affection, at least to that state of the nervous system termed "nervousness," the "hysterical temperament," "great sensitiveness," &c., and the leading characteristic of which is an exalted susceptibility of all impressions, or, as Dr. Laycock terms it, *affectibility*, and a refinement of the feelings and intellect, such, that the individual is hardly equal to the ordinary wear and tear of life.

That the hygiène of the young female, as now practised amongst the middle and higher classes of society, conduces to this precocious development of the reproductive organs, is, we think, tolerably well established. The influence of climate, and other circumstances, on the age of first menstruation has been made a subject of statistical inquiry by numerous observers. Dr. Tilt has collected the data supplied by them, and arranged them in tabular forms; and he finds that temperature has really much influence. Thus, while the mean age of first menstruation is 12½ years at Calcutta, it is 14¾ in London, and 16 at Copenhagen. Dr. Brierre de Boismont found that town and country life made a difference; for at Paris the mean age is 14 years, 6 months, while in the country it is 4 months more. Luxury and poverty make even a greater difference than town and country; for the same inquirer ascertained the following facts as to the mean age at which this important event first took place in different classes of society:

| | YEARS. | MONTHS. |
|---|---|---|
| In 171 of the poor . . . . . . . . . . | 14 | 10 |
| " 135 of the well-to-do working classes . . | 14 | 5 |
| " 53 daughters of the rich and noble . . | 13 | 8 |

Dr. Tilt attributes this difference to the higher temperature of the mansions of the rich, by which civilization "brings about the precocious fructification of the human germ, in the same way that the gardener in the hot-house does that of the vegetable tribes." This appears to us to limit the circumstance to one set of causes: doubtless, the less exposure to the weather may have its influence, but the high feeding, and the excitation of the nervous system by dancing, music, &c. will have its effect. It is quite a mistake to suppose that these stimuli cannot act on the ovaria and through them on the development of the body, without being sexually felt, or without exciting distinct emotions. In many lower animals, and not less in man, it is certain that they will have a mere *automatic* unfelt influence. Besides, if we understand Dr. Tilt rightly, it is the solar heat to which he attributes this maturating property, "that imponderable compound which is heat, light, and magnetism," &c. Now we can scarcely think that coal-fires would have this effect.

But the evolution of the reproductive organs has only begun with the first appearance of the periodic flow; for several successive years, the work has to progress, and the perfect woman to be developed. During this period the education in public schools goes on, and by the age of seventeen the young lady is introduced into society. It is then that the strain upon the constitutional powers generally, and the reproductive system specially, commences. But it may be, that vicious habits have been already acquired, and the ovaria have been unduly excited by Lesbian pleasures. If such have been the case, then the round of conventional usages to which she is now introduced act with much more intensity upon the organism. The polka and the waltz bring her into exciting contact with the other sex; and we think that an amorously-constituted girl cannot engage in these dances, in the indecent manner, at least, in which some men perform their share of the dance—without an undue excitement of the sexual feelings—injurious to the health, if not to the morals. Indeed, such excitation is sometimes obvious. It is much to be regretted that individuals should be found in society, who take advantage of the love of the sex for dancing, and press young girls to their bosom, or otherwise manipulate so as to shock the feelings of modest women. That such instances do occur has come within our own knowledge, for we have known ladies assign acts like these as the reason for refusing to dance with individuals of the other sex; and we suspect that the dances we allude to, and indeed all others in which the two sexes come into close personal contact, will be more and more rarely danced, if this abuse be not checked by the emphatic denunciation of society.

Dancing, ballad-music, excessive devotion to needlework, reading of love-stories, (which all novels are,) promenade-concerts, and balls, are all more or less excitants of the sexual feelings—add to these, the intimate and unrestrained social intercourse with the other sex which English society, more than any other in Europe, permits its young women to enjoy; and we have a number of sexual stimuli, amply sufficient to account for many of the diseases to which we have alluded. How far all this is necessary to the great end of woman's life, we do not pretend to say. The subject has, however, attracted the attention of moralists of late years; and we trust that the legitimate use of the stimuli we refer to will be substituted for the abuse. We wish to be understood, however, as repudiating altogether the notion that these stimuli are indulged by English women more than others of the sex in Europe; on the contrary, we gather from extensive statistical data, that the women of Great Britain are remarkable for their chastity. Our remarks are, indeed, rather directed against practices which undoubtedly tend to lower our countrywomen from their pre-eminence in this respect.

The clothing of the sex is an important point of inquiry; for there can be no doubt that the universal denunciation of *tight lacing* by the medical profession, is founded on a too general abuse of stays and corsets. It is a remarkable circumstance, that the most extraordinary and absurd costumes may become admired and generally prevalent if *fashionable*, that is to say, worn by elegant and richly-dressed women. In reality, it is the native grace and elegance of the leaders of the *mode* which renders the costume pleasing. The difficulty with regard to the use of stays is not in convincing the sex that they are hurtful—indeed, many ladies take the greatest care not to be girded unpleasantly by them—but rather in the invention of a style of dress which shall maintain bodily warmth and display the figure so as not to render it massy and rigid. The *fits* of corsets aim at something like the natural outline of the figure; and the appendage to the posterior torso, known as a "bustle," is evidently intended to imitate nature in *her* natural proportions. Independently of the circumstance, that a mass of padding laid across the loins cannot but exercise an injurious influence on the ovaria and uterus, the thing is objectionable from its palpable absurdity in being set where it is, as a substitute for, or addition to, the graceful contour of the hips. What is wanted is, a style of dress so adapted to the figure that it shall drape it gracefully, and half hide, half disclose, its undulating contours. Such a dress is worthy the study of the painter, and might fittingly occupy the mind of even a man of artistic genius. An invention of this kind would not only relieve the female figure from a pressure, alike destructive to health, temper, and charms, but also set woman free to take those gymnastic exercises which can alone develop true grace of form and movement, while they add health and strength both of body and mind. The free unrestrained combination of the muscles and groups of muscles, and the performance of every innate or instinctive action, can never be attained while the due flexion, extension, and rotation of the trunk and arms are limited by straps and corsets, however elastic and yielding. It is, indeed, this restraint which prevents the sex engaging in various games appropriate to their sex; or, when engaged, prevents them giving full play to the entire muscular system. If once a drapery adapted to display a graceful form became the fashion, the exercises and pursuits suitable to its development would be as ardently insisted on by the mother, and as diligently practised by the daughter, as the corset is now studied.

Now we have ample proof that it is possible to clothe the human figure, especially that of the female, so that it shall be a beautiful object. And although the recorded examples of this are to be found almost exclusively in tropical, or the southern portions of temperate, climes, yet it cannot be denied that very fine examples of feminine grace and symmetry may be found amongst the lowest classes of all European nations; or, anyhow, amongst the peasant girls of Great Britain and Ireland. The ancient Greek dress might, we think, be easily adapted to the European female in the present advanced stage of our manufactures, especially in elastic woven tissues. The graceful tunic, descending in folds to the feet, was supported across the shoulders by clasps or jewelled buttons, and made to show the outline of the bust, by a zone or belt across the thorax. The *ricinium* fitted over this, not unlike the modern polka jacket, and the *mantle* hung gracefully, when required, over all. Instead of cutting the figure into two halves, like the modern European lady, and making herself look unlike anything else in creation, the Greek woman used two zones, one beneath the bust, as just stated, and the other across the abdomen, so that the natural graceful outline of the torso was maintained, and support given where it is often most required, that is, in the woman who has borne children. We need hardly observe that stiff petticoats belong to the same class of things as bustles, and that garters and girding belts and straps are all objectionable, and destructive to beauty of form. The exposure of the neck and upper part of the thorax is, we believe, more common in England than in any other part of Europe. The custom probably arises from the superior development and colour of the bust in the English woman. The French have always expressed their admiration of it. Niceron relates a story how, in 1610, one Thomas Dampster having married an Englishwoman in London, brought her to Paris, and how, as she walked in the streets with her husband, dressed *à l'Anglaise,* displaying the most beautiful throat and neck, and shoulders of dazzling whiteness, the glorious apparition attracted so large a crowd of admirers, that the pair ran a risk of suffocation, and had to take refuge in the first house.

The exposure of the throat and bust depends much on the age and complexion; for a woman of taste will hardly exhibit a yellow wrinkled neck; and as it is precisely that portion of the body which is beyond the reach of cosmetics, if not passable it is shut out from vision. A French writer (Barthe) touches gracefully on this point. Dulaç was a perfumer, and Laudumier a dentist of Paris, of the last century.

"On peut se donner des yeux doux,

Se faire une petite bouche:

Toutes n'ont pas, ainsi que vous,

Ces roses dont l'éclat me touche;

Telle chez Dulac va payer

Son teint, qui doit tourner vos têtes;

Telle, au besoin, chez Laudumier

A de belles dents toutes prêter;

Le sien—mais je n'ose appuyer;

Passons plus bas; pied ridicule

Bien à l'étroit dans une mule

Peut nous paraître un piéd léger,

*Mais pour le cou*, ma foi, mesdames,

Je défie un sénat des femmes

De pouvoir jamais le changer."

The question as to the use of cosmetics, and, indeed, as to the *dress* and *decoration* of woman generally, must be decided on general principles. The Creator has provided that all the most attractive traits in woman's person shall indicate either *moral* or *physical fitness* for her duties as woman. The brilliant lips, the transparent clear complexion, indicate health; the whole body, when in its fully developed form and functions, indicates perfect capacity and fitness to reproduce the species—to produce not only offspring, but a healthy race; and, with the *physical capacity*, the requisite *moral feelings* and *sentiments*. It is the sum total of these external indices of sexual fitness which makes up the charming *tout ensemble* in the eye of man. Hence, vigorous health and perfect corporeal development are always the most attractive. Whatever is requisite to attain these, whether in dress, in personal cares, or diet, it is becoming a woman to seek after; but whatever is adopted, with sexual objects in view, to give the *appearance of* only, or to hide the deficiency in, any of these characteristics, is unbecoming. The practice is an untruth, whether it be adopted to hide defects in the form or complexion. Various kinds of corsets, and various styles of dress, are constantly used with the innocent intention of adding new beauty to existing charms, or of hiding what would offend *all* eyes. This is justifiable, for it is part of woman's nature to make herself as pleasing as possible to her own, as well as the other sex; it is only when some hideous disease is concealed from a lover that the practice is criminal; or, when the art of adornment is directed to the excitation of the mere instinct of sexual congress in man. Whenever, in any nation or people, the women have made it their great object thus to acquire or display *meretricious charms*, they have lost in moral beauty what they have gained in external appearance. The charm of modesty, truthfulness, and simplicity, is lost to the character, and the morals themselves have become insensibly depraved. The history of woman amongst the Greeks and Romans, and amongst many modern Asiatic nations, affords ample proof that the general use of mere cosmetics, of meretricious ornaments, and of exciting modes of dress, degrades the character of woman, reduces her to a mere slave to the sensual pleasures of man, and corrupts all the finer feelings of human nature. We would, however, permit the aged woman to wear false teeth, for they are useful; or even to wear false hair, for at least, no cheat is intended to be practised on a lover; but there is a beauty even in age, and the gray hair, toothless gums, and wrinkled skin of the loving, gentle, intellectual matron, have a charm and dignity of their own, with which paint, false ringlets, and false teeth, are quite incompatible.

Feminine purity, therefore, and the dignity of the feminine character are not incompatible with the practice of all those arts which will add beauty to the person, but cannot co-exist, or at least are endangered, by those which are directed solely to the excitation of the sexual instinct in man. Elegance and taste in dress and ornament; a due attention to personal hygiène, especially the daily use of the bath; temperance in all enjoyments; free exercise in the open air, especially gymnastics, directed to the due development of the figure; moderate cultivation of the feelings and of the intellect; an intelligent regard for religious duties; these are legitimate means of rendering the person and manner attractive. Anything *meretricious* (in the strict etymological sense of the word) —any mode of dress, any ornament or cosmetic, which the prostitute peculiarly adapts to her vocation—can only sully and degrade.

These observations lead us to a consideration of woman's *social* position. Now whatever may be said of the *rights* of woman, it is her allotted *duty* to marry and bear children. It is obvious, however, that in Christian and highly civilized nations, it is not possible for every woman to fulfil her mission; for although the numbers of each sex living at one time are nearly equal, yet since many *men* do not marry, many women *cannot*, and are, therefore, doomed to celibacy perforce, wherever polygamy is forbidden. There were living in June, 1841, in England, nearly four millions of women, aged from 15 to 45; of these, $1\frac{3}{4}$ million were married, leaving more than two millions unmarried, of whom only *one in seventeen* is married annually. Of these two millions, thus cut off from the great ends of their existence, the lot is indeed very various, but the greater proportion must necessarily have to labour for their bread, in one way or the other. Herein is, indeed, a great problem to solve. The order of nature is, that the woman shall be devoted to the cares of maternity and the domestic duties of life; the order of society is, that millions shall have no husband, and therefore, legitimately, no children. The order of nature seems to be, that as maternal cares occupy the woman exclusively, her sustenance and protection, and the sustenance and protection of her children, should devolve upon man; the order of society deprives millions of women of a mate and a protector. Under these circumstances, how does she fare?

The inquirer need not look far for an answer, if he be a dweller in a populous city. Everywhere woman is competing with man for the means of living; and, with an instinctive selfishness, man has sadly limited the sphere of her labour. There are so many things that it is not *proper* for a woman to do, or so many that she *cannot* do, or so many that she cannot do *so well* as man, that alas! she is too often driven to an avocation in which it is not possible for man to compete with her, or else must accept the fearful alternative of the bitterest poverty and privation.

The pursuits of the prostitute seem to date from an early period of history. We have an early instance recorded in Scripture, in such terms as would indicate that, in the time of the Patriarchs, it was no uncommon avocation for a woman. Prostitution seems to have prevailed almost universally, indeed; in every nation, at every age of the world. In some it has received the sanction of religion, in others of public opinion, in others of the state; while in others, to be found guilty of it was certain death. So widely have ideas differed upon a point, upon which both nature and religion would appear to coincide. Perhaps by nothing is the female man so degraded as by being made the minister to the mere brutish instincts of the male man. Every trace of human dignity and moral grandeur must needs be swept away by so gross a perversion of the sex from its divine ends. And yet, even amidst this wreck of all that is sweet and glorious in her nature, something of the true feminine nature still survives and flashes forth amidst the darkness. Parent du Chatelet, one of the most philanthropic men of the age, devoted much time and labour to an inquiry into the condition of these unfortunate women in Paris—for unfortunate they truly are. The picture he draws of their psychological condition is extremely interesting. Abroad, and before the world, they are impudent, boasting, and reckless; if you obtain their confidence and learn the true state of their feelings, it is found that they are weighed down by a sense of their degradation. The sight of virtuous women and mothers of families is insupportable; they envy them while they insult them. Nor are their feelings blunted to anything like the extent that is supposed. Du Chatelet overheard a party one day talking in the ward of the hospital, when one exclaimed, "what a charming sky; God is indeed good to send us such beautiful weather! He treats us better than we deserve;" and all the ward answered at once, "That is very true." He also observes, that the sole consideration of not having any one to love them, and no worthy object of their affections, drives them often to madness. The natural sentiment of modesty does not appear to be banished even from the most abandoned; if a stranger enters the lock-up or dormitory of the police office at the moment they are dressing, they immediately cross their arms over the chest to hide the bosom. Those who associate with soldiers often tattoo themselves, and the characters they have imprinted on the upper part of the arm, below the mammæ, or on the abdomen, have all reference to their affections. Initials will be tattooed, with the motto *pour la vie*, or simply P. L. V. often placed between two flowers, or two hearts interlaced and pierced by an arrow. It is remarkable that the names differ with the age; if the *fille de pavé* be young, it is that of a man—if she be of "a certain age," it is that of a woman, and in a particular spot, namely, between the pubes and umbilicus. The *pour la vie* is but a conventional phrase, for the inscriptions are often numerous. Du Chatelet counted thirty on the bust only of a girl in the hospital of the prison La Force, and there were more on other parts of the body. Sometimes they are obliterated, when a small scar is left. Du Chatelet counted fifteen such scars on the arms, throat, and chest of a girl under 25 years of age.

This inconstancy is not to be charged to the unfortunate girl so much as to her lover. It appears, indeed, that the instinct to love, in all its relations, is very strong. They are singularly kind and charitable to each other when in distress; if one be about to become a mother, she is an object of the warmest interest; during her accouchement they load her with attentions; they will wash her and the infant, take care of their linen, and almost quarrel about the child. If they have children, there are no better mothers in the world; and so far from neglecting their morals (if they be girls), they carefully watch over their virtue and provide for their advancement in life. Nothing indeed is so pleasing to them as to become a mother.

But the question that most concerns us is the determining cause of prostitution; and it is of great importance to observe, that in by far the majority of cases it is followed as a trade or profession. Du Chatelet made inquiries into the causes in 5183 instances, with the following results:

| | |
|---|---|
| Absolute starvation and want | 1441 |
| Orphanage, or expulsion from home | 1255 |
| To maintain aged and infirm parents | 37 |
| " orphans, sisters, nieces, &c. | 29 |
| Widows, or deserted wives, for the maintenance of their children | 23 |
| Women from the country seeking a livelihood | 280 |
| Brought to Paris from the country by soldiers, students, &c., and then deserted | 404 |
| Servants seduced by their masters | 289 |
| Kept mistresses, who had lost their lovers | 1425 |

It is remarkable how much, in these cases, the unhappy women were more sinned against than sinning; few, Du Chatelet remarks, can be found who have followed the avocation from mere licentiousness; although some were so young as ten years, some so old as sixty-five.

This state of things is due, as Du Chatelet observes, to the competition we have already alluded to, and to the intrusion of men into employments, which it would be more honourable to them to give up to the other sex. Is it not shameful, he asks, to see numbers of men, in the prime of life, passing an effeminate existence in cafés, shops, and warehouses; or washing up pots, and smoothing linen? There can be no doubt that women are adapted to many employments now filled by men. Modelling, painting, designing, and the fine arts generally, might be practised by them with much greater success, if their intellect received an early training. The teaching of youth, and the care of the sick, are other employments to which they are eminently adapted. They might be more extensively employed as *sage-femmes*. Domestic servitude might be rendered available to a more extensive class of women than it is at present, by a more careful education, and by a training directed expressly to fit woman for her duties. At present there is a lamentable want of facilities for the acquisition of what may be termed women's handicrafts. Cooks, nurses, parlour-maids, housemaids, nurserymaids, &c., are left to pick up their knowledge as they can, at much risk to themselves, and often with considerable loss to their employers. We hope, however, the time is not far distant, when provision will be made for the more effectual instruction of young women in occupations suitable to their intellectual and bodily powers, and so the greater number of the 2,000,000 of unmarried females in England will not be left dependent upon their friends, or jostled out of the ranks by manly competitors for effeminate employments, as they now are.

The *problem* of *increase* of *population* can have no accurate or satisfactory solution, without a due estimate of the psychological relations of the sex to society. The enforced celibacy to which we have just adverted may be and is triumphantly quoted as an illustration of the doctrine of natural checks, arising out of moral considerations. There can be no doubt of the truth of the general statement, namely, that the increase in the middle and higher classes, by births, is barely sufficient to supply the loss caused in those classes by deaths. This is dependent upon three causes. Firstly, the marriages are not contracted at so early a period as in the lower classes, consequently there are fewer births in each family. Secondly, the earlier development of the reproductive functions in those classes, to which we have already alluded, appears to be not without an influence on their fecundity; but be this as it may, it is a fact that there are fewer children to each married pair than in the lower classes. And thirdly, many of the women in this class, influenced by prudential or conventional motives, do not marry at all. Now it is obvious, we think, that the check in the increase of population is applied where it is least likely to be beneficial, for the vacancies in the ranks of humanity, caused by the less prolificness of the higher classes, are rapidly filled up from the ranks below them. Thus the salutary influences which the educated woman might exert as a wife and mother are lost to society, and replaced by the influence of the ungentle, uneducated, and untrained. We think it is also worth consideration whether, if due attention were paid to the *physique* of the middle and higher classes, and a higher standard of corporeal health established, the higher grade of intellectual development, and the finer sensibilities they have acquired, might not be handed down from generation to generation, and the progressive improvement and elevation of the *whole* race secured. The etiquette of social rank acts as an insuperable bar, in many cases, to the *diffusion* of these acquired powers of mind throughout the ranks below; women and men remaining single, rather than contracting an alliance with persons of inferior rank; or else, in the case of the men, transmitting parental peculiarities to *illegitimate* offspring, who, coming into the world in the same low social rank as their mothers, with considerable natural powers of mind—perhaps with the pride and ambition of the father—enter into life with those powers untrained, and so constitute the most dangerous class of society.

But prudential considerations have been enforced in some states by law upon all classes, and what has been the result? The average age at which marriage has been contracted has been deferred, but not the age of intercourse of the sexes; and hence an increase in the number of illegitimate children, an increase in prostitution, and a wider diffusion of all those various evils which result from a disregard of the irrevocable laws of nature. Thus, in Bavaria, 20½ per cent. of all the children born are illegitimate; in Saxony, 15 per cent.; in Wurtemberg and Austria, 11½ per cent.; in Hanover and Denmark, 9½ per cent.; in France and Prussia, 7 per cent.; in England, Belgium, Sweden, and Norway, 6½ per cent.; while in Sardinia, only 2 per cent. are illegitimate.* But the births of illegitimate children only indicate a part of the injury done to society by the prudential check; all so born will not be registered; many perish *in utero;* many conjugal as well as licentious connexions are systematically rendered infertile; and many men lose their true manly character, by unnatural stimulation of the reproductive organs. The courage of the male man disappears, and is replaced by the dissimulation characteristic of the sex, a trait of character which may be traced in a *whole profession*, as well as in individuals living a solitary life in college, or in the world.

To the psychologist, who desires to see the human species improve

* Sixth Annual Report of the Registrar-General, p. xxxv.

progressively in all that constitutes the glory of man as a created being, this destruction of life, and these checks to increase and improvement in the most highly developed races, must constitute a matter of deep regret. The remedies for these evils are within reach, although certainly difficult of attainment. One of these is a wider sphere of industrial occupation for woman, whether married or single, so that marriage and children may be rendered desirable by being rendered less burdensome to the man. Some means for extending this sphere we have hinted at; and we think that if education and intellect be made to constitute the main requisites for successful feminine industry, the poorly-pensioned gentlewoman will no longer esteem honest industry to be derogatory to her dignity, as too many at present do; for many of these suffer pinching poverty in silence; they "cannot dig, to beg they are ashamed." A second remedy is a higher moral and intellectual training for the whole sex, and especially an extension of the scheme of education, so that it shall include less of the merely ornamental, and more of the useful branches of knowledge. The golden rule should be applied to girls which is so generally applied to boys, namely, that every woman should be taught some useful art adapted to her faculties and social position, and by which she may be able, if circumstances require, either to add to her husband's means, or to maintain herself and children. That a much wider field of intellectual and industrial enterprise is open to woman than she is at present permitted to occupy is, we think, amply demonstrated by us in our views as to her providential and social position. A third remedy would be the wider promulgation of the doctrine, that the man is imperfect, and cannot be well developed either bodily or mentally—can attain to no true symmetry and beauty—who is without a mate.

And now, having discoursed of so many and such varied topics touching woman in her psychological relations, not, we trust, without interesting our readers, we may be permitted cordially to re-echo and repeat the sentiments expressed by Dr. Laycock in the following passage, contained in the dedication of his work to Sir James Clark:

"The subject upon which I have written is too dignified and interesting to require any other introduction to the world than its own merits. In support of this proposition, the philanthropist might observe, that all the best feelings of humanity should urge us to continued effort for the welfare of the sex; the political economist might advance, that the power of a people is indissolubly connected with the physical well-being of its females; and the moral philosopher might show, that the moral and intellectual greatness of Britain is based on the domestic virtues, pure morals, and elevated sentiments of its women."

We repeat, we cordially concur with Dr. Laycock in the expression of these sentiments, and it is because we feel their weight we have thus discussed at length one of the most interesting topics of the age—the subject of woman in her psychological relations.

The preceding observations are based upon two works.* The able production of Dr. Laycock, of York, bears internal evidence that its author is a scholar, a philosopher, and an accomplished physician. He has, in writing the work, spared no pains to render his treatise every way worthy of a place in the libraries of the members of the medical profession. He wishes it, indeed, to be considered as a second edition, revised and improved, of an essay which had already appeared, as a series of articles on hysteria, in the Edinburgh Medical and Surgical Journal for 1838-39. The work is divided into three parts; the first comprises the special physiology of woman, in her corporeal and mental relations: the second treats of the general pathology and principles of treatment of the nervous diseases to which she is peculiarly liable during a certain period of life; and the third is devoted to the consideration of each special form of disease in detail. The plan of his inquiry is inductive, and is founded upon general principles deduced from a large number of facts. He shows that the greater number of the diseases of women, termed "spinal," "hysterical," &c., have their seat in the nervous system; that, as a class, they are almost peculiar to the sex; that women of susceptible nervous system are more liable than others; and that the diseases under consideration occur in the sex during that period of life only in which the reproductive organs perform their functions.

Starting from these principles, and taking them as his guide, Dr. Laycock progressively unfolds his subject. He firstly defines the reproductive organs, and shows that the ovaria are the all-important constituents, since it is upon these that the special feminine characteristics are dependent. He shows the relations of these organs to distant and remote parts of the frame; their sympathetic action on the cerebro-spinal axis in particular, from the brain downwards; and the monthly and occasional changes which they excite—including the doctrines of periodic recurrence of morbid phenomena. Dr. Laycock next proceeds to investigate the mental and corporeal peculiarities of woman, with reference especially to the affectibility of her nervous system, as compared with that of man; her physical conformation, the constitution of her vascular system, and the composition of her blood. Then he concludes that the diseases to which she is liable have their origin in an aggravation of the peculiar affectibility of her nervous system, *primarily*, by changes in

* A Treatise on the Nervous Diseases of Women, comprising an Inquiry into the Nature, Causes, and Treatment of Spinal and Hysterical Disorders. By Thomas Laycock, M.D., Physician to the York Dispensary, &c.

The Morbid Emotions of Women; their Origin, Tendencies and Treatment. By Walter Johnson, M.B., formerly Medical Tutor, Guy's Hospital.

the composition of the blood, and *secondarily*, by the direct and indirect influence of the ovaria. Hence Dr. Laycock objects to the terms "hysterical" and "nervous," as applied to these functional disorders, but recommends the use of the term *neurœmic* (since adopted by some writers) as applicable to the whole class of diseases of the nervous system, in which morbidly constituted blood reacts upon a morbidly constituted nervous system. In the second part, after a special consideration of the physiology of the nervous system, Dr. Laycock considers the *pathology* of his subject, and elucidates it as he proceeds by the light of physiology. Thus he traces out an important analogy between the diseases of general development—taking dentition as a guide—and the diseases which depend more obviously upon the monthly periodic change in the sex, incidentally noting, at the same time, the effects of bad methods of education and training on the development of the nervous system of woman in general, and of the reproductive system and its functions in particular. A chapter is next devoted to the influence of morbid conditions of the blood on the nervous system, and the causation of diseases seated therein by excessive blood-letting and other exhausting agencies, depraving the blood by poisons—including febrile, metallic, and animal and vegetable poisons—and certain *excreta* retained in the blood, especially urea, and the *materies morbi* of gout. Under the latter head, Dr. Laycock takes occasion to show the intimate connexion between the hereditary gouty constitution and the whole class of nervous diseases, not only in woman but in man also. Lastly, he notices the influence of the passions and emotions. Dr. Laycock closes the second part, pointing out the relations of the nervous system in general to its diseases, and lays down the general principles of treatment.

In the third part, the various affections to which young women are liable, and which have hitherto defied all arrangement, are classified *physiologically*, that is to say, according to the relations which the organs or tissues affected, or the portions of the nervous system that are the seat of disease, bear to the reproductive organs—in the way previously established. He is thus enabled to pass in review, in connected series and in relation to each other, each and every one of the varied and puzzling disorders which have been hitherto treated under widely different heads, and ascribed to as widely different causes; laying down the rules for the diagnosis and treatment of each.

The book by Dr. Johnson is a very different production—its title is altogether a misnomer; for the whole work, with the exception of a few pages, consists of a consideration, such as it is, of some of the nervous diseases considered by Dr. Laycock in his systematic work; all the reference that is made to the "morbid emotions" is incidental, unless

[illegible] MEDICAL LIBRARY
WASHINGTON, D. C. U. S. A.

"apoplexy," "epilepsy,' '"tetanus," "catalepsy," "tarantulism," "American spider," "tic-tic or hicum," and other nosological matters and things, can (contrary to the ordinary use of language) be properly termed "morbid emotions." This free use of language in a novel sense is paralleled by a singular assertion touching the bibliography of his subject; and the medical reader is not a little startled by the following round declaration.

"Considering, therefore, that although the peculiarities manifested by young females, as a class, have been frequently dwelt upon by medical authorities, yet that these peculiarities have always been discussed as isolated problems, and never brought together nor systematized—considering, I say, that no attempt has been made to embrace them all in a general view, I have thought it not unprofitable to collect a variety of cases, illustrative of each affection that presented itself to me, to form a basis upon which, at some future period, a comprehensive theory may be constructed," &c. *Preface*, p. vi.

The problems of woman's nature, we hardly need to state, have been too interesting in all ages to have suffered that neglect which Dr. Johnson, with juvenile precipitancy, claims to be the first to remedy. The *Opus Coronatum* of Landouzy, (which, Dr. Johnson is not aware contains a *catalogue raisonnée* of 373 cases, principally from continental authors,) is a sufficient proof of the importance attached to the morbid conditions of the sex by physicians and philosophers, and we venture to say, that a *catalogue raisonnée* of works on woman—her natural history, her physiology, her psychology, and her pathology—would occupy at least a sheet of our journal.

We are at a loss to reconcile this reiterated assertion of Dr. Johnson's, both with actual facts and with the evidence which his book exhibits, that although he thus ignores the existence of Dr. Laycock's work, he has not only read Dr. Laycock's essays in the Edinburgh Medical and Surgical Journal, but has appropriated some of the results of that physician's literary researches. Those essays consist of a selection of cases similar to Dr. Johnson's, but much more varied and extensive; selected, too, with a special reference to an analysis of their phenomena; and with the definite object of embracing them "all in a general view," and discussing them in their mutual relations, and *not* "as isolated problems." We repeat, that we are quite at a loss to reconcile with these facts the bold assertion made by Dr. Johnson which we have quoted above—and we think that Dr. Johnson will experience a similar difficulty.

